# Etiology-Based Comparative Evaluation of Toxic Hepatitis in a Tertiary Referral Cohort: Drug-Induced, Herb-Induced, Mushroom Poisoning, and Other Toxic Exposures

**DOI:** 10.3390/medicina62071290

**Published:** 2026-07-03

**Authors:** Suheda Rumeysa Osmanlioglu-Dag, Nurullah Dag, Sami Akbulut, Muhsin Murat Muhip Harputluoglu, Sezai Yılmaz

**Affiliations:** 1 Department of Pharmaceutical Botany, Faculty of Pharmacy, Inonu University, 44280 Malatya, Türkiye; 2 Department of Radiology, Faculty of Medicine, Inonu University, 44280 Malatya, Türkiye; 3 Department of Surgery and Liver Transplantation, Faculty of Medicine, Inonu University, 44280 Malatya, Türkiye; akbulutsami@gmail.com (S.A.); sezai.yilmaz@inonu.edu.tr (S.Y.); 4 Department of Biostatistics and Medical Informatics, Faculty of Medicine, Inonu University, 44280 Malatya, Türkiye; 5 Department of Gastroenterology, Faculty of Medicine, Inonu University, 44280 Malatya, Türkiye; murat.harputluoglu@inonu.edu.tr

**Keywords:** drug-induced liver injury, DILI, hepatotoxicity, herbal products, HILI, mushroom poisoning, toxic hepatitis

## Abstract

*Background and Objectives*: Toxic hepatitis may result from drugs, herbal products, mushroom exposure, or other suspected toxic agents. This study aimed to compare the clinical, laboratory, severity-related, and outcome characteristics of toxic hepatitis according to suspected etiology. *Materials and Methods*: This retrospective observational study included 175 patients diagnosed with toxic hepatitis between January 2012 and March 2026 at a tertiary referral center with an active liver transplantation program. Patients were categorized into five groups: drug-induced liver injury (DILI) (*n* = 97), herb-induced liver injury (HILI) (*n* = 17), mushroom poisoning (*n* = 24), suspected causative agent exposure (*n* = 15), and unavailable etiological data (*n* = 22). Clinical outcomes, laboratory parameters, injury patterns, severity grades, and MELD-Na scores were analyzed. *Results:* Etiological groups differed significantly in demographic, hematological, biochemical, coagulation, bilirubin, ammonia, MELD-Na, and R-ratio parameters. Mushroom poisoning showed the most pronounced hepatocellular injury profile, with higher median values for AST, ALT, INR, ammonia, MELD-Na, and R ratio: 4856 U/L, 4996 U/L, 5.0, 585 µg/dL, 30, and 78.3, respectively. Recovery was most frequent in the HILI group (94.1%). In multivariable logistic regression analysis, older age (OR = 1.066, 95% CI: 1.03–1.11), lower INR (OR = 0.205, 95% CI: 0.08–0.51), and higher albumin level (OR = 4.255, 95% CI: 1.55–11.63) were independently associated with recovery. *Conclusions*: Toxic hepatitis showed distinct clinical and biochemical patterns according to etiology. In this selected tertiary referral-center cohort, mushroom poisoning was associated with pronounced hepatocellular injury and frequent liver transplantation, whereas HILI showed a more favorable short-term course. These findings should be interpreted in light of referral bias and the overrepresentation of severe cases.

## 1. Introduction

Toxic hepatitis represents acute liver injury caused by exogenous hepatotoxic agents, including pharmaceutical drugs, herbal products and dietary supplements, toxic mushrooms, and other suspected chemical or biological exposures [1,2,3]. Although these various etiologies have overlapping clinical manifestations, they may differ in terms of biochemical patterns, severity, prognosis and need for liver transplantation [4,5,6]. Accurate etiological classification and comparative characterization of these distinct forms of toxic hepatitis are essential for optimizing clinical management, guiding causality assessment, and informing public health strategies.

Drug-induced liver injury (DILI) is the most extensively studied form of toxic hepatitis and represents a leading cause of acute liver failure in Western countries [5,6]. DILI is clinically relevant because it accounts for approximately 10% of all cases of acute hepatitis and up to 50% of acute liver failure cases in some registries [5,7,8]. The condition is classified into intrinsic DILI, which is dose-dependent and predictable (e.g., acetaminophen hepatotoxicity), and idiosyncratic DILI, which is unpredictable, not dose-dependent, and mediated by immune or metabolic susceptibility factors [1,9]. Diagnosis of DILI relies on careful exclusion of other causes of liver injury, detailed medication history, and temporal relationship between drug exposure and liver injury onset [1,3]. The European Association for the Study of the Liver (EASL) Clinical Practice Guidelines provide a standardized framework for DILI diagnosis, emphasizing the importance of causality assessment using validated tools such as the Roussel Uclaf Causality Assessment Method (RUCAM) [1,3]. Severity grading is typically based on biochemical patterns (hepatocellular, cholestatic, or mixed), peak transaminase and bilirubin levels, and the presence of coagulopathy or encephalopathy [1,9]. Outcome evaluation includes assessment of mortality, need for liver transplantation, and long-term sequelae such as chronic liver disease [9].

Herb-induced liver injury (HILI) has gained increasing recognition as a significant cause of toxic hepatitis worldwide [2,10,11,12,13,14,15]. Large registry analyses have documented over 12,000 published HILI cases globally, with substantial regional variation in implicated products [11,12]. Diagnostic challenges in HILI are considerable and stem from the heterogeneous composition of herbal formulations, contamination with heavy metals or pharmaceutical adulterants, use of polyherbal preparations containing multiple botanicals, inconsistent product labeling that obscures ingredient identity and dosage, and incomplete patient disclosure of herbal and supplement use during clinical encounters [2,10,13]. Unlike conventional pharmaceuticals, herbal products are often marketed without rigorous premarket safety evaluation, and batch-to-batch variability further complicates causality assessment [10,13,14]. Application of RUCAM and other causality tools to HILI is challenging because rechallenge data are rarely available, and the precise hepatotoxic constituent within a complex mixture may remain unidentified [11,12].

Mushroom poisoning-related hepatotoxicity, particularly from amatoxin-containing species such as *Amanita phalloides*, represents a distinct and often life-threatening form of toxic hepatitis [16,17]. Amatoxins inhibit RNA polymerase II, leading to severe hepatocellular injury that typically manifests 6 to 24 h after ingestion with gastrointestinal symptoms, followed by a latent period and subsequent fulminant hepatic failure [16]. Acute liver failure develops in a substantial proportion of patients with amatoxin poisoning, and mortality remains high despite supportive care and extracorporeal detoxification methods [16,17,18,19]. Prognostic assessment in mushroom poisoning relies on clinical criteria and biochemical markers [18,19]. Urgent liver transplantation is often required for survival in patients who develop severe coagulopathy, encephalopathy, or multi-organ failure [16,18,19].

Comparative data among different etiological groups in toxic hepatitis remain limited. Most available studies focus on a single exposure category, such as DILI, HILI, or mushroom poisoning, rather than evaluating these entities within a unified clinical framework. A comparative approach may help clarify whether distinct etiologies are associated with different clinical characteristics and disease trajectories. Therefore, this study aimed to compare the laboratory findings, liver injury severity, prognostic markers, and clinical outcomes of patients with toxic hepatitis according to etiology, including DILI, HILI, mushroom poisoning, and suspected or not available causative agent.

## 2. Materials and Methods

### 2.1. Study Design

This retrospective observational cohort study was conducted at a tertiary referral center with an active liver transplantation program. The study included patients diagnosed with toxic hepatitis between January 2012 and March 2026. Clinical, laboratory, prognostic, and transplant assessment data generated during routine clinical care in the index hospitalization were retrospectively analyzed. The study protocol was approved by the Inonu University Institutional Review Board for Non-Interventional Studies (Approval Number: 9744; Date: 7 April 2026). Because anonymized data were analyzed retrospectively, the requirement for individual informed consent was waived.

The study was designed to evaluate the etiological spectrum, biochemical injury pattern, severity profile, prognostic assessment, and short-term clinical outcomes of patients with toxic hepatitis. In this study, toxic hepatitis was used as an umbrella term for acute liver injury temporally and clinically compatible with exposure to a potentially hepatotoxic agent, including prescription or over-the-counter drugs, herbal products or dietary supplements, toxic mushrooms, or other suspected toxic exposures, after exclusion of competing causes of liver injury [20,21,22,23,24,25,26].

### 2.2. Patient Selection

Patients were eligible for inclusion if they fulfilled all of the following criteria: (1) acute liver injury compatible with toxic hepatitis, defined by at least one of the following biochemical thresholds: alanine aminotransferase (ALT) or aspartate aminotransferase (AST) > 5 times the upper limit of normal (ULN), alkaline phosphatase (ALP) > 2 times the ULN, total bilirubin ≥ 2.5 mg/dL or international normalized ratio (INR) ≥ 1.5 in the presence of elevated liver enzymes, or ALT/AST > 3 times the ULN together with total bilirubin > 2 times the ULN; (2) a documented toxic exposure, referral diagnosis of toxic hepatitis, or clinical presentation considered compatible with toxic hepatitis after exclusion of competing etiologies; (3) availability of admission laboratory variables required for biochemical injury pattern classification and routine prognostic assessment; and (4) a documented clinical outcome, categorized as recovery, liver transplantation, or death [3,21,22,23].

Patients were excluded if diagnosis or outcome could not be verified, key laboratory data were missing, or advanced chronic liver disease/decompensated cirrhosis could independently explain the presentation. Alternative causes of acute liver injury were excluded according to the standard clinical work-up used in our center, including viral hepatitis serology, autoimmune markers when indicated, abdominal imaging for biliary obstruction, Doppler ultrasonography for vascular or ischemic causes, and clinical assessment for sepsis-related or other relevant etiologies [21,22,23]. In transplanted patients, the endpoint was liver transplantation; post-transplant outcomes were beyond the scope of this study.

### 2.3. Etiological Classification

Patients were classified according to the presumed causative exposure into five categories: DILI, HILI, mushroom poisoning, suspected causative agent, and unavailable etiological data. Etiological classification was based on the recorded exposure history, latency between exposure and liver injury, clinical course, biochemical phenotype, and exclusion of alternative etiologies [20,21,22,23,24,25,26].

For DILI and HILI, etiological attribution was made by the hepatology team at the time of diagnosis using a structured clinical approach consistent with the RUCAM framework. This approach included assessment of exposure chronology, latency, biochemical injury pattern, clinical course after discontinuation of the suspected agent, concomitant medications or herbal/dietary supplement use, and exclusion of competing causes of liver injury [4,24,27]. Because formal itemized RUCAM scores were not routinely recorded in the electronic medical records, retrospective numerical RUCAM scoring could not be reliably performed for all patients. Therefore, RUCAM was considered a diagnostic reference framework but was not analyzed as a quantitative study variable.

Mushroom poisoning was classified separately on the basis of a witnessed or clinically credible wild mushroom ingestion history, compatible latency, gastrointestinal prodrome when present, subsequent hepatotoxic course, and exclusion of competing causes of acute liver injury [4,20,24].

The suspected causative exposure group included patients with a documented or clinically credible history of exposure to potentially hepatotoxic non-pharmaceutical and non-herbal substances that could not be categorized as DILI, HILI, or mushroom poisoning. These exposures included chemical substances, synthetic or recreational toxic agents, explosive or combustible material ingestion, agricultural or household chemicals, and other non-medical toxic agents when documented in the clinical records.

The unavailable exposure data group (NA) included patients who fulfilled the clinical and biochemical criteria for toxic hepatitis and in whom competing etiologies, including viral hepatitis, autoimmune liver disease, biliary obstruction, ischemic hepatitis, sepsis-related liver injury, and identifiable drug-, herbal product-, or mushroom-related causes, were evaluated and not identified, but no specific hepatotoxic exposure could be documented from the available medical records. Therefore, unavailable exposure data was treated as an indeterminate exposure-data category rather than as a distinct biological etiology and was handled separately in etiological subgroup analyses.

### 2.4. Data Collection

Clinical and laboratory data were retrospectively extracted from institutional electronic medical records using a standardized data abstraction form. The recorded variables included age, sex, etiological exposure category, suspected causative agent when available, clinical outcome, transplant status, transplant type when applicable, and admission laboratory parameters. Laboratory variables included white blood cell count, lymphocyte count, neutrophil count, hemoglobin, platelet count, AST, ALT, ALP, gamma-glutamyl transferase (GGT), INR, total bilirubin, direct bilirubin, albumin, creatinine, ammonia, and sodium. For biochemical injury pattern classification, severity grading, and prognostic assessment, admission values were used. Admission values were defined as the first available laboratory results obtained at the study center before liver transplantation or another definitive outcome event. When more than one result was available on the day of admission, the value closest to the first clinical evaluation at the study center was selected. The normal reference ranges for laboratory parameters (Table S1) and a representative radiological image (Figure S1) are provided in the Supplementary Material.

### 2.5. Liver Injury Pattern

The biochemical pattern of liver injury was determined using the R ratio [1,21,26]: R ratio = (ALT/ALT ULN) ÷ (ALP/ALP ULN). An R ratio ≥ 5 was classified as hepatocellular injury, an R ratio > 2 and <5 as mixed injury, and an R ratio ≤ 2 as cholestatic injury [1,21,26]. This standardized approach was applied across all etiological groups to allow comparison of biochemical injury phenotypes in DILI, HILI, mushroom poisoning, suspected causative agent, and unavailable etiological data.

### 2.6. Hy’s Law Assessment

Hy’s law-related biochemical criteria were assessed to identify cases with hepatocellular injury accompanied by clinically relevant hyperbilirubinemia. Because the cohort included non-DILI etiologies, Hy’s law was used as a biochemical risk phenotype rather than as evidence of drug causality. Standard Hy’s law biochemical positivity was defined as ALT or AST > 3 times the ULN together with total bilirubin > 2 times the ULN, after exclusion of an alternative explanation for hyperbilirubinemia. A stricter classical Hy’s law definition was also evaluated by additionally requiring ALP < 2 times the ULN. This secondary definition was used to reduce potential misclassification of patients in whom hyperbilirubinemia could be primarily explained by a cholestatic pattern of liver injury [1,21].

### 2.7. Liver Injury Severity Grading

The severity of liver injury was graded using a DILIN-derived five-level liver injury severity classification. This grading system was applied as a descriptive severity framework and not as a causality assessment tool. This distinction was necessary because the cohort included toxic mushroom-related hepatotoxicity and indeterminate toxic hepatitis in addition to DILI and HILI. Grade 1 indicated mild liver injury, defined as enzyme elevation without jaundice or coagulopathy. Grade 2 indicated moderate liver injury, defined as enzyme elevation with total bilirubin ≥ 2.5 mg/dL and/or INR ≥ 1.5. Grade 3 indicated moderate-to-severe liver injury requiring hospitalization or prolongation of hospitalization attributable to toxic hepatitis. Grade 4 indicated severe liver injury with jaundice and evidence of hepatic failure, such as coagulopathy, ascites, encephalopathy, or other organ failure attributable to liver injury. Grade 5 indicated death or liver transplantation attributable to toxic hepatitis. Because grade 5 incorporates liver transplantation and death, this variable was used for descriptive severity reporting and was not entered as an independent predictor in analyses where liver transplantation or death constituted the outcome [3,28].

### 2.8. Prognostic Scores

The Model for End-Stage Liver Disease incorporating serum sodium (MELD-Na) score was routinely assessed in adult patients using admission INR, total bilirubin, creatinine, and sodium values according to the standard allocation-based formula [29]. Conventional lower-bound constraints for bilirubin, creatinine, and INR, and standard sodium bounding, were applied during calculation. For patients younger than 12 years, the Pediatric End-Stage Liver Disease (PELD) score was assessed when the required variables were available [30].

### 2.9. Clinical Outcomes

The primary clinical outcome was the final clinical status related to the toxic hepatitis episode. Outcomes were categorized as recovery, liver transplantation, or death. Recovery was defined as clinical improvement without liver transplantation and without death attributable to toxic hepatitis. Liver transplantation was defined as transplantation performed for toxic hepatitis-related acute liver failure or severe toxic liver injury after multidisciplinary transplant assessment [31]. Death was defined as mortality attributable to toxic hepatitis or toxic hepatitis-related acute liver failure. For additional analyses, liver transplantation and death were combined as an adverse clinical outcome. This composite endpoint was used to evaluate factors associated with clinically severe disease progression.

### 2.10. Statistical Analysis

Statistical analyses were performed using IBM SPSS version 25.0 Statistics for Windows, (IBM Corp., Armonk, NY, USA). ROC curve analysis and graphical visualizations were performed using GraphPad Prism, version 10.6.1 (GraphPad Software, Boston, MA, USA). Continuous variables were summarized as median with interquartile range (IQR), and categorical variables were presented as frequencies and percentages.

Continuous variables were compared using the Kruskal–Wallis test for comparisons involving more than two independent groups and the Mann–Whitney U test for two-group comparisons. When the overall Kruskal–Wallis test was significant, post hoc pairwise comparisons were performed using Bonferroni adjustment. Categorical variables were compared using the Pearson chi-square test, Fisher’s exact test, or the Fisher–Freeman–Halton exact test, as appropriate according to expected cell counts and table dimensions. For categorical variables with a significant overall comparison, Bonferroni-adjusted pairwise column proportion tests were used for post hoc pairwise comparisons.

An exploratory multivariable binary logistic regression analysis was performed to evaluate factors independently associated with recovery. Variables entered into the final model were age, AST, INR, total bilirubin, albumin, ammonia, and creatinine. Odds ratios (ORs) were reported with 95% confidence intervals (CIs). Model discrimination was assessed using receiver operating characteristic (ROC) curve analysis based on the predicted probabilities generated from the final logistic regression model. The area under the ROC curve (AUC) with 95% CI was reported. Adjusted ORs and 95% CIs were displayed using a forest plot. Missing data were not imputed, and analyses were performed using available cases. A two-sided p value below 0.05 was considered statistically significant.

## 3. Results

A total of 175 patients with toxic hepatitis were included. Etiological classification identified 97 patients with DILI, 17 with HILI, 24 with mushroom poisoning, 15 with suspected causative exposure, and 22 with NA.

### 3.1. Comparisons Across Etiological Groups

Clinical and laboratory characteristics differed significantly across etiological groups (Table 1 and Table 2). Age and sex distribution differed significantly among groups, and the median age was lower in the suspected causative agent and NA groups.

Mushroom poisoning showed a distinct inflammatory and hepatocellular injury profile, characterized by higher WBC and neutrophil counts, lower lymphocyte counts, markedly elevated AST and ALT levels, higher R-ratio values, and higher INR and ammonia levels. DILI and NA groups showed higher bilirubin levels, whereas HILI was generally associated with lower INR, lower MELD-Na scores, and better preserved albumin levels. Hepatocellular injury was the predominant biochemical pattern across all etiological groups.

Disease severity and clinical outcomes also differed significantly by etiology (Table 2). Severe liver injury was more frequent in DILI, mushroom poisoning, suspected causative agent cases, and NA, whereas mild-to-moderate injury predominated in HILI. HILI was associated with the highest recovery rate and the lowest transplantation rate. In contrast, liver transplantation was more frequent in DILI, mushroom poisoning-related hepatotoxicity, suspected causative agent cases, and NA. All patients in the NA group underwent transplantation, while mortality occurred only in the DILI group.

### 3.2. Mushroom Poisoning Versus Other Causes

Compared with non-mushroom toxic hepatitis, mushroom poisoning-related hepatotoxicity showed a more pronounced hepatocellular and systemic injury profile (Table 3 and Table 4). Patients with mushroom poisoning had significantly higher WBC and neutrophil counts, AST, ALT, INR, ammonia, MELD-Na scores, and R-ratio values, whereas lymphocyte count, GGT, total bilirubin, and direct bilirubin levels were significantly lower.

No significant differences were observed between mushroom poisoning and other causes in categorical clinical characteristics, including sex, transplant type, biochemical pattern of liver injury, Hy’s law positivity, DILIN-derived severity grade, or clinical outcome (Table 4).

### 3.3. Clinical Outcome-Specific Findings

Clinical and laboratory variables differed significantly according to outcome status (Table 5 and Table 6). Patients who underwent liver transplantation were younger and showed a more severe biochemical and clinical profile than recovered patients, including higher WBC and neutrophil counts, AST, ALP, INR, bilirubin, ammonia, and MELD-Na scores, together with lower lymphocyte counts, hemoglobin, GGT, and albumin levels.

Categorical analyses showed that Hy’s law positivity and severe DILIN-derived liver injury grade were more frequent among patients who underwent liver transplantation, whereas moderate injury was more common among recovered patients. Etiological distribution also differed by outcome, with HILI being overrepresented among recovered patients. Mortality was limited to four patients, all in the DILI group.

When patients were further dichotomized as recovery versus liver transplantation/death, the same pattern was observed (Table 7 and Table 8). The liver transplantation/death group had higher markers of systemic and hepatic injury, higher MELD-Na scores, more frequent Hy’s law positivity, and a predominance of severe DILIN-derived liver injury grade.

### 3.4. Multivariable Logistic Regression and Model Discrimination

**Figure 1:**
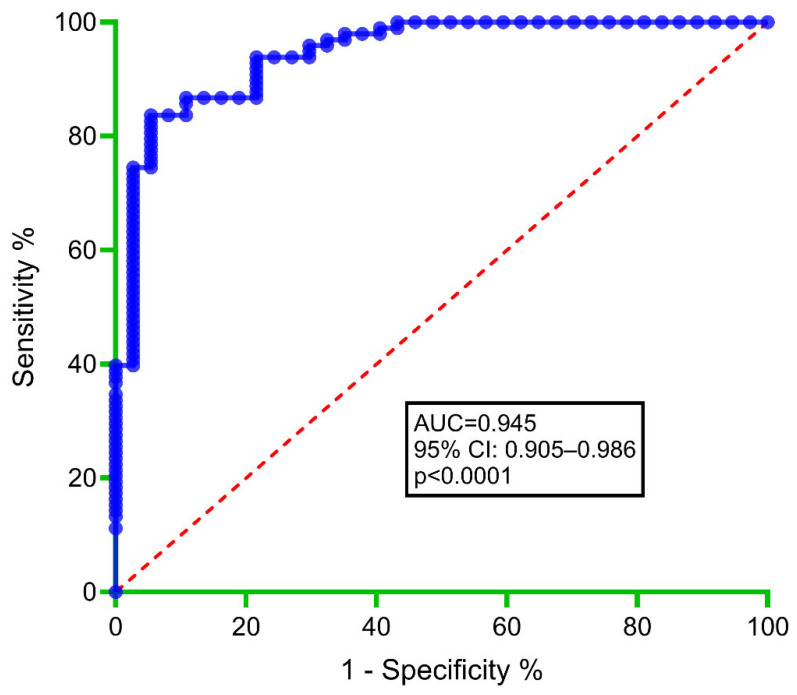
ROC curve of the multivariable logistic regression model for distinguishing patients with LT/death from those who recovered. The model demonstrated excellent discriminatory performance, with an area under the curve of 0.945 (95% CI: 0.905–0.986; *p* < 0.0001).

**Figure 2:**
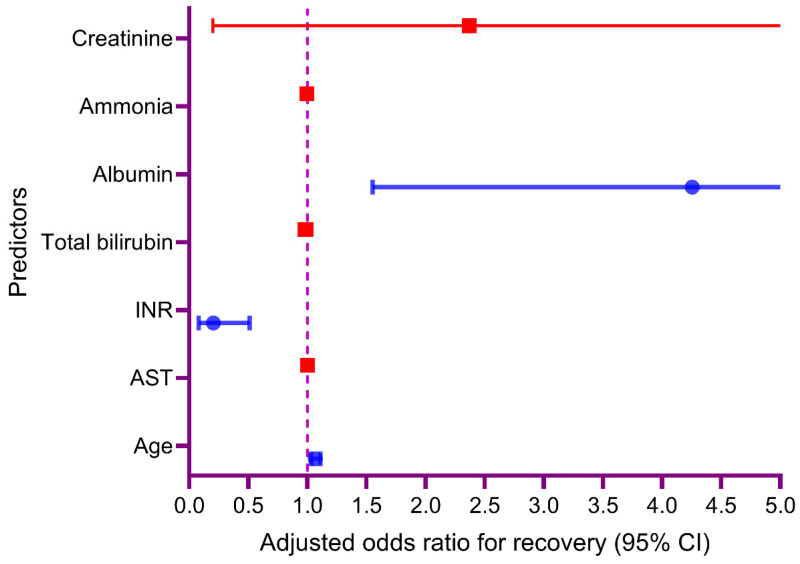
Forest plot showing adjusted odds ratios for recovery derived from the multivariable logistic regression model. Odds ratios greater than 1 indicate a higher likelihood of recovery, whereas odds ratios less than 1 indicate a lower likelihood of recovery. Horizontal bars represent 95% confidence intervals, and the vertical dashed line indicates an odds ratio of 1.

Table 9 shows the exploratory multivariable logistic regression model evaluating factors associated with recovery rather than LT/death. Older age, lower INR, and higher albumin level were independently associated with recovery. Each one-year increase in age increased the odds of recovery by 1.07-fold (OR = 1.066, 95% CI: 1.03–1.11; *p* = 0.001). Each one-unit increase in INR reduced the odds of recovery (OR = 0.205, 95% CI: 0.08–0.51; *p* = 0.001), whereas each one-unit increase in albumin increased the odds of recovery by 4.26-fold (OR = 4.255, 95% CI: 1.55–11.63; *p* = 0.005). Figure 1 demonstrates that the multivariable model had excellent discriminatory performance for distinguishing recovery from LT/death, with an area under the receiver operating characteristic curve of 0.945 (95% CI: 0.905–0.986; *p* < 0.0001). Figure 2 presents the adjusted odds ratios and 95% confidence intervals for the variables included in the multivariable logistic regression model.

## 4. Discussion

In this retrospective cohort of patients with toxic hepatitis managed at a tertiary referral center with an active liver transplantation program, etiological groups differed in severity, biochemical profile, and outcome. DILI was the largest etiological category, while mushroom poisoning was characterized by marked hepatocellular injury, higher INR and ammonia levels, and frequent liver transplantation. HILI showed a more favorable short-term course. Lower INR, higher albumin, and older age were independently associated with recovery. Given the transplant-capable referral setting, the high transplantation rates should be interpreted as reflecting a selected cohort enriched for severe cases rather than population-level estimates.

DILI was the largest etiological group in the present cohort and showed a clinically severe profile, with predominantly hepatocellular injury, marked bilirubin elevation, and a high rate of liver transplantation. This finding is consistent with previous studies and reviews describing DILI as a broad clinical entity with hepatocellular, cholestatic, or mixed biochemical patterns and a clinical course ranging from mild liver enzyme elevation to acute liver failure [21,22,23,32,33,34,35,36]. However, the clinical severity observed in our DILI group was higher than that reported in unselected DILI cohorts. In the review by Leise et al. [37], prospectively collected DILIN data showed death in 8% and liver transplantation in 2% of idiosyncratic DILI cases; the same review also noted that idiosyncratic DILI accounts for approximately 11–13% of acute liver failure cases in the United States. In contrast, 56.7% of our DILI patients underwent liver transplantation and 4.1% died. This discrepancy most likely reflects the transplant-center setting of the present study, where mild or self-limited DILI cases are underrepresented and severe cases are preferentially referred. The high frequency of Hy’s law positivity, hyperbilirubinemia, and severe DILIN-derived injury grade in our DILI group further supports the prognostic relevance of bilirubin elevation and impaired hepatic function in clinically severe DILI. Thus, our findings likely represent the severe end of the DILI spectrum rather than population-level estimates of DILI outcome.

Herbal product-related liver injury showed a more favorable course in the present cohort than the other major etiological groups. Most patients with HILI recovered without transplantation, and this group had lower INR and MELD-Na values, suggesting less prominent hepatic functional impairment at presentation. This finding should be interpreted cautiously, because previous studies have shown that HILI can occasionally progress to acute liver failure, liver transplantation, or death [23,38]. In a multicenter Taiwanese cohort, Huang et al. [38] reported that patients with HILI had higher rates of jaundice, coagulopathy, encephalopathy, acute liver failure, and mortality than those with conventional DILI. Similarly, Soares et al. [39] found that 7.6% of published HILI cases were fatal or required liver transplantation. Therefore, the favorable outcome observed in our HILI group should not be interpreted as evidence that herbal products are intrinsically safe or that HILI is generally mild. Rather, it may reflect the small number of HILI cases, the specific products used, earlier discontinuation, lower toxicity of the implicated preparations, or referral characteristics of this cohort.

The diagnostic uncertainty surrounding HILI also remains relevant to the interpretation of our findings. Unlike regulated pharmaceutical agents, herbal products may contain multiple botanicals, variable concentrations of active compounds, undeclared ingredients, contaminants, or adulterants [40]. Halegoua-DeMarzio et al. [41] and Frenzel et al. [42] emphasized that HILI is largely a diagnosis of exclusion and that product quality, mislabeling, contamination, and multi-ingredient formulations may complicate causality assessment. In addition, patient disclosure may be incomplete because many individuals do not perceive herbal products or supplements as medications. Botanical verification, DNA barcoding, and complementary authentication approaches may improve identification of implicated plant materials or mislabeled products, although their routine clinical use remains limited [43,44]. These considerations support careful exposure history, exclusion of alternative etiologies, and, when feasible, botanical or chemical verification in HILI cases. They also underscore the potential value of collaboration between hepatologists, clinical toxicologists, and pharmaceutical botanists in the evaluation of suspected plant-related toxic hepatitis.

Mushroom poisoning-related hepatotoxicity showed the most severe hepatocellular injury profile in the present cohort, with markedly higher aminotransferase levels, R-ratio values, INR, ammonia, and MELD-Na scores, as well as a high rate of liver transplantation. This pattern is consistent with the recognized clinical course of severe amatoxin-related poisoning, in which an initial gastrointestinal phase may be followed by delayed hepatocellular injury, coagulopathy, acute liver failure, and, in selected cases, liver transplantation [24,25,45,46,47]. Previous series and meta-analytic data have shown that most mushroom poisonings are self-limited, but a small subset progresses to fulminant hepatic failure or death, particularly when symptom onset is delayed or amatoxin exposure is suspected [48,49]. In this context, the high transplantation rate observed in our mushroom poisoning group most likely reflects referral of severe cases to a transplant-capable center rather than the expected outcome of mushroom poisoning in the general population. When compared with published international series evaluating amatoxin poisoning, the mushroom poisoning group in the present study appears to represent a highly severe subgroup. In the study by Ganzert et al., prognostic assessment for liver transplantation after amatoxin intoxication was based on serial prothrombin index and serum creatinine values [50]. Escudié et al. reported that fatal intoxication, defined as death or liver transplantation, occurred in 30% of patients with Amanita phalloides poisoning, and suggested that a prothrombin index below 10% of normal from day 4 after ingestion may be a strong indicator for emergency liver transplantation [16]. In our cohort, the mushroom poisoning group had median AST and ALT values of 4856 U/L and 4996 U/L, respectively, and a median INR of 5.0, indicating marked hepatocellular and synthetic dysfunction. The liver transplantation rate in this group was 75.0%, while no in-hospital death was observed. This pattern should be interpreted in the context of a selected tertiary referral cohort with an active liver transplantation program and the availability of living-donor liver transplantation, rather than as a population-level outcome estimate for mushroom poisoning.

The absence of formal species-level identification remains an important limitation in the interpretation of mushroom-related toxic hepatitis. This is clinically relevant because mushroom poisoning includes several toxin-mediated syndromes, whereas severe hepatocellular injury, acute liver failure, and the need for transplantation are particularly associated with amatoxin-related exposures [45,46]. Since species confirmation is often not feasible in routine emergency practice, early risk stratification usually depends on exposure history, latency from ingestion to symptom onset, clinical course, and serial laboratory changes rather than definitive mycological confirmation [51,52]. Thus, the lack of formal mycological verification in our cohort should be regarded not only as a methodological limitation, but also as a reflection of real-world diagnostic uncertainty in mushroom poisoning-related hepatotoxicity.

The suspected causative exposure and unavailable exposure data groups reflect a common real-world challenge in the evaluation of toxic hepatitis. In some patients, exposure to a non-pharmaceutical toxic agent is clinically documented, whereas in others the clinical picture is compatible with toxic liver injury but no specific hepatotoxic exposure can be identified despite exclusion of competing causes. Incomplete histories, limited documentation, polypharmacy, undisclosed supplement use, or lack of confirmatory testing may contribute to this uncertainty. The high transplantation rate observed in the unavailable exposure data group should therefore be interpreted with caution and may reflect the transplant-center setting, severe presentation at referral, incomplete etiological ascertainment, or an unrecognized underlying cause rather than the effect of a distinct biological etiology. Clinically, however, the absence of a defined causative agent should not reduce concern when biochemical and functional parameters indicate severe liver injury.

Outcome-based analyses underscored the clinical importance of early hepatic functional assessment. Patients who underwent liver transplantation or died had higher INR, bilirubin, ammonia, MELD-Na scores, inflammatory markers, and DILIN-derived severity grades than those who recovered. In the exploratory multivariable analysis, lower INR and higher albumin remained independently associated with recovery, indicating that preserved hepatic synthetic function was closely related to favorable outcome. Together with ammonia and MELD-Na, these parameters reflect the extent of hepatic functional impairment and are directly relevant to transplant assessment in severe acute liver injury [29,31].

Hy’s law positivity was also more frequent among patients who underwent liver transplantation or died. In this cohort, however, Hy’s law should be interpreted as a biochemical risk phenotype rather than evidence of drug causality, because the study included mushroom poisoning, HILI, suspected causative agent cases, and unavailable etiologies in addition to classical DILI. The association between older age and recovery should also be interpreted cautiously, as it may reflect cohort composition and etiological distribution rather than a direct protective effect. Although the multivariable model showed strong internal discrimination, it remains exploratory and requires external validation before clinical use.

### Strengths and Limitations

The main strengths of this study include the evaluation of a relatively large toxic hepatitis cohort from a tertiary referral center with an active liver transplantation program, the use of a unified institutional database, standardized etiological classification, systematic assessment of biochemical injury patterns, and clearly defined outcome classification. In addition, the study provides a comparative analysis of DILI, HILI, mushroom poisoning, suspected toxic exposures, and unavailable etiological data within the same clinical framework.

However, several limitations should be noted. First, the retrospective single-center design may have introduced referral and selection bias, and mild or self-limited cases managed outside the transplant setting were likely underrepresented. Therefore, the high transplantation rates observed in this cohort should not be interpreted as population-level estimates. Second, exposure histories relied on medical records and patient or family reporting, and formal botanical, mycological, or toxicological confirmation was not routinely available. Third, although DILI and HILI diagnoses were established by hepatologists using a structured causality approach consistent with RUCAM principles, formal itemized RUCAM scores were not routinely recorded; therefore, numerical RUCAM categories could not be analyzed. Finally, the unavailable exposure data group represents an indeterminate exposure-data category and may include patients with incomplete histories, undocumented exposures, or unrecognized alternative etiologies. Accordingly, the inclusion of this group may have introduced heterogeneity and potential residual confounding, particularly in outcome-related analyses and in the exploratory multivariable model.

## 5. Conclusions

In conclusion, toxic hepatitis differed according to suspected etiology in terms of biochemical profile, clinical severity, and outcome. In this selected tertiary referral cohort, mushroom poisoning was associated with pronounced hepatocellular injury and frequent liver transplantation, whereas HILI had a more favorable acute clinical profile. These findings highlight the importance of early etiological assessment, close monitoring of hepatic functional parameters, and multidisciplinary collaboration among hepatologists, clinical toxicologists, and pharmaceutical botanists, particularly when herbal product- or mushroom-related toxic hepatitis is suspected.

## Figures and Tables

**Table 1:** Comparison of continuous clinical and laboratory variables across etiological groups.

Variable	DILI	HILI	Mushroom Poisoning	Suspected Causative Agent	NA	*p*
Age	34 (26–50) ^c^	47 (33–59) ^c^	38 (15–51) ^b,c^	5 (3–20) ^a^	13 (2–44) ^a,b^	<0.001
WBC	8 (6–10) ^a,b^	7 (6–8) ^a^	10 (8–18) ^b^	7.0 (5.0–11) ^a,b^	9 (8–11) ^a,b^	0.004
Lymphocyte	1.6 (1.2–2.3) ^b^	1.8 (1.8–1.9) ^b^	0.6 (0.5–0.9) ^a^	1.8 (1.5–3.7) ^b^	1.3 (1.0–1.9) ^a,b^	<0.001
Neutrophil	5.3 (3.5–7.9) ^b^	4.2 (3.0–5.3) ^a,b^	8.9 (5.9–17.2) ^c^	3.0 (2.4–4.9) ^a^	6.8 (3.8–9.3) ^b,c^	<0.001
Hemoglobin	12.6 (11.2–14.0) ^b^	14.5 (13.6–15.7) ^a^	12.4 (10.5–13.5) ^b^	11.2 (10.8–14.4) ^a,b^	12.2 (10.4–12.9) ^b^	0.008
Platelet	206 (141–288) ^a^	221 (202–287) ^a^	189 (131–244) ^a^	254 (207–325) ^a^	200 (108–266) ^a^	0.190
AST	1001 (458–1740) ^a,c^	687 (400–797) ^a^	4856 (2412–9162) ^b^	1503 (654–2259) ^a,c^	2161 (891–5984) ^b,c^	<0.001
ALT	958 (522–1971) ^a^	1043 (670–1261) ^a,c^	4996 (3311–6707) ^b^	1115 (875–2028) ^a,c^	2496 (836–5517) ^b,c^	<0.001
ALP	192 (138–238) ^a,c^	101 (66–155) ^a^	166 (108–234) ^a,c^	275 (210–497) ^b^	248 (134–403) ^b,c^	<0.001
GGT	102 (62–178) ^a^	62 (22–542) ^a,b^	54 (30–68) ^b^	48 (30–75) ^b^	60 (50–136) ^a,b^	<0.001
INR	2.1 (1.5–3.2) ^b^	1.3 (1.2–1.4) ^a^	5.0 (3.6–9.3) ^c^	3.0 (1.8–3.5) ^b,c^	3.9 (2.5–5.9) ^c^	<0.001
Total bilirubin	16.0 (7.9–24.0) ^b^	3.0 (1.0–4.2) ^a^	4.2 (2.5–5.7) ^a^	9.0 (7.3–18.0) ^a,b^	13.8 (5.4–21.0) ^b^	<0.001
Direct bilirubin	10.0 (5.0–16.0) ^b^	2.0 (0.4–2.7) ^a^	2.8 (1.1–3.6) ^a^	6.0 (4.0–13.0) ^b^	9.4 (3.1–12.0) ^b^	<0.001
Albumin	3.0 (2.5–3.5) ^a^	4.0 (3.5–4.1) ^b^	3.5 (3.3–3.6) ^b^	3.4 (3.0–3.8) ^a,b^	2.9 (2.5–3.2) ^a^	<0.001
Ammonia	147 (111–230) ^b^	116 (97–259) ^b^	585 (186–1075) ^a^	151 (105–195) ^b^	218 (151–487) ^a,b^	<0.001
Creatinine	0.7 (0.6–0.8) ^b^	0.7 (0.6–0.9) ^b^	0.7 (0.6–1.4) ^b^	0.4 (0.3–0.7) ^a^	0.6 (0.5–0.8) ^a,b^	<0.001
Sodium	137 (135–139) ^a^	139 (136–140) ^a^	138 (134–140) ^a^	136 (135–138) ^a^	136 (133–139) ^a^	0.620
MELD Na	27 (20–32) ^b^	15 (8–16) ^a^	30 (27–40) ^b,c^	27 (23–27) ^a,b,c^	35 (30–37) ^c^	<0.001
PELD score	26 (22–28) ^a^	—	41 (26–41) ^a^	28 (18–31) ^a^	29 (27–31) ^a^	0.494
R ratio	14.7 (8.4–26.8) ^a^	29.5 (12.2–44.6) ^a^	78.3 (60.2–150.8) ^b^	16.8 (5.3–27.4) ^a^	28.5 (14.8–41.3) ^a^	<0.001

Data are presented as median (Q1–Q3). *p* values were calculated using the Kruskal–Wallis test. Superscript letters indicate Bonferroni-adjusted post hoc pairwise comparisons. Values sharing at least one superscript letter are not significantly different.

**Table 2:** Comparison of categorical clinical characteristics across etiological groups.

Variable	Category	DILI	HILI	Mushroom Poisoning	Suspected Causative Agent	NA	*p*
Sex	Male	38 (39.2)	9 (52.9)	6 (25.0)	11 (73.3)	9 (40.9)	0.039
	Female	59 (60.8)	8 (47.1)	18 (75.0)	4 (26.7)	13 (59.1)	
LT Type	LDLT	37 (67.3)	1 (100)	12 (66.7)	8 (72.7)	19 (86.4)	0.477
	DDLT	18 (32.7)	0 (0)	6 (33.3)	3 (27.3)	3 (13.6)	
Pattern of liver injury n (%)	Hepatocellular	79 (81.4)	17 (100)	24 (100)	13 (86.7)	20 (90.9	0.240
	Cholestatic	11 (11.3)	0 (0)	0 (0)	1 (6.7)	2 (9.1)	
	Mixed	7 (7.2)	0 (0)	0 (0)	1 (6.7)	0 (0)	
Hy’s law n (%)	Negative	25 (25.8)	7 (41.2)	6 (25.0)	2 (13.3)	2 (9.1	0.162
	Positive	72 (74.2)	10 (58.8)	18 (75.0)	13 (86.7)	20 (90.9)	
Classical Hy’s law n (%)	Negative	37 (38.1)	10 (58.8)	11 (45.8)	10 (66.7)	11 (50)	0.183
	Positive	60 (61.9)	7 (41.2)	13 (54.2)	5 (33.3)	11 (50)	
DILIN-derived liver injury severity grade, n (%)	Mild	4 (4.1)	6 (35.3)	2 (8.3)	0 (0)	0 (0)	<0.001
	Moderate	29 (29.9)	10 (58.8)	4 (16.7)	2 (13.3)	1 (4.5)	
	Severe	64 (66.0)	1 (5.9)	18 (75.0)	13 (86.7)	21 (95.5)	
Outcome, n (%)	Recovered	38 (39.2)	16 (94.1)	6 (25.0)	4 (26.7)	0 (0)	<0.001
	LT	55 (56.7)	1 (5.9)	18 (75.0)	11 (73.3)	22 (100)	
	Death	4 (4.1)	0 (0)	0 (0)	0 (0)	0 (0)	

**Table 3:** Comparison of continuous clinical and laboratory variables between mushroom poisoning and non-mushroom toxic hepatitis.

Variables	Mushroom Poisoning (*n* = 24)	Other Causes (*n* = 151)	*p*
Age	38 (15–51)	33 (20–47)	0.518
WBC	10 (8–18)	8 (6–11)	0.006
Lymphocyte	0.6 (0.5–0.9)	1.6 (1.2–2.4)	<0.001
Neutrophil	8.9 (5.9–17.2)	5.0 (3.3–7.5)	<0.001
Hemoglobin	12.4 (10.5–13.5)	12.7 (11.0–14.3)	0.310
Platelet	189 (131–244)	221 (146–287)	0.092
AST	4856 (2412–9162)	1032 (537–2045)	<0.001
ALT	4996 (3311–6707)	1031 (571–2226)	<0.001
ALP	166 (108–234)	194 (131–268)	0.125
GGT	54 (30–68)	86 (50–174)	0.002
INR	5.1 (3.6–9.3)	2.2 (1.4–3.4)	<0.001
Total bilirubin	4.2 (2.5–5.7)	13.6 (5.4–22.0)	<0.001
Direct bilirubin	2.8 (1.1–3.6)	9.0 (3.1–14.0)	<0.001
Albumin	3.6 (3.3–3.6)	3.1 (2.6–3.5)	0.002
Ammonia	585 (186–1075)	160 (111–240)	<0.001
Creatinine	0.7 (0.6–1.4)	0.7 (0.6–0.8)	0.044
Sodium	138 (134–140)	137 (135–139)	0.862
MELD Na	30 (27–40)	26 (19–32)	0.025
PELD score	41 (26–41)	29 (22–31)	0.276
R ratio	78.3 (60.2–150.8)	17.1 (9.3–32.0)	<0.001

**Table 4:** Comparison of categorical clinical characteristics between mushroom poisoning and non-mushroom toxic hepatitis.

Variable	Categories	Mushroom Poisoning (*n* = 24)	Other Causes (*n* = 151)	*p*
Sex	Male	6 (25.0)	67 (44.4)	0.074
	Female	18 (75.0)	84 (55.6)	
LT Type	LDLT	12 (66.7)	65 (73.0)	0.583
	DDLT	6 (33.3)	24 (27.0)	
Pattern of liver injury *n* (%)	Hepatocellular	24 (100)	129 (85.4)	0.135
	Cholestatic	0 (0.0)	14 (9.3)	
	Mixed	0 (0.0)	8 (5.3)	
Hy’s law *n* (%)	Negative	6 (25.0)	36 (23.8)	0.902
	Positive	18 (75.0)	115 (76.2)	
Classical Hy’s law *n* (%)	Negative	11 (45.8)	68 (45.0)	0.942
	Positive	13 (54.2)	83 (55.0)	
DILIN-derived liver injury severity grade, *n* (%)	Mild	2 (8.3)	10 (6.6)	0.511
	Moderate	4 (16.7)	42 (27.8)	
	Severe	18 (75.0)	99 (65.6)	
Outcomes	Recovered	6 (25.0)	58 (38.4)	0.283
	LT	18 (75.0)	89 (58.9)	
	Death	0 (0.0)	4 (2.6)	

**Table 5:** Comparison of continuous clinical and laboratory variables according to clinical outcome.

Variables	Recovered (*n* = 64)	LT (*n* = 107)	Death (*n* = 4)	*p*
Age	40 (33–55) ^b^	24 (9–42) ^a^	60 (59–62) ^b^	<0.001
WBC	7 (6–9) ^a^	9 (7–14) ^b^	8 (7–8) ^a,b^	<0.001
Lymphocyte	1.8 (1.4–2.3) ^b^	1.3 (0.8–1.9) ^a^	1.7 (0.8–3.1) ^a,b^	0.006
Neutrophil	4.6 (3.5–5.4) ^a^	7.2 (3.7–9.8) ^b^	4.5 (3.8–5.3) ^a,b^	0.001
Hemoglobin	13.6 (12.3–15.0) ^b^	12.0 (10.3–13.2) ^a^	13.7 (12.0–14.4) ^a,b^	<0.001
Platelet	221 (170–270) ^a^	202 (126–271) ^a^	233 (135–323) ^a^	0.618
AST	878 (519–1651) ^a^	1661 (787–3394) ^b^	991 (409–3056) ^a,b^	0.001
ALT	1057 (649–2090) ^a^	1437 (606–3994) ^a^	1053 (543–1875) ^a^	0.216
ALP	163 (102–218) ^a^	199 (134–282) ^b^	258 (198–344) ^a,b^	0.010
GGT	129 (56–225) ^b^	64 (43–113) ^a^	192 (138–905) ^b^	<0.001
INR	1.4 (1.2–1.8) ^a^	3.5 (2.4–4.9) ^b^	2.2 (1.7–2.8) ^a,b^	<0.001
Total bilirubin	6.3 (1.9–16.5) ^a^	13.6 (5.7–22.0) ^b^	17.8 (8.3–30.0) ^a,b^	0.001
Direct bilirubin	4.7 (1.0–11.5) ^a^	8.0 (3.5–13.0) ^b^	13.4 (6.4–21.0) ^a,b^	0.005
Albumin	3.5 (2.9–3.8) ^b^	3.1 (2.5–3.5) ^a^	2.5 (2.2–2.7) ^a^	<0.001
Ammonia	106 (88–186) ^a^	201 (141–431) ^b^	189 (126–297) ^a,b^	<0.001
Creatinine	0.7 (0.6–0.8) ^b^	0.6 (0.5–0.8) ^a^	0.6 (0.6–0.8) ^a,b^	0.035
Sodium	138 (136–139) ^a^	136 (134–140) ^a^	137 (129–142) ^a^	0.922
MELD Na	19 (14–25) ^a^	32 (27–36) ^b^	27 (26–31) ^a,b^	<0.001
PELD score	—	29 (23–31)	—	—
R ratio	20.9 (11.7–40.3) ^a^	22.9 (9.3–51.6) ^a^	17.2 (5.3–28.2) ^a^	0.642

Data are presented as median (Q1–Q3). *p* values were calculated using the Kruskal–Wallis test. Superscript letters indicate Bonferroni-adjusted post hoc pairwise comparisons. Values sharing at least one superscript letter are not significantly different.

**Table 6:** Comparison of categorical clinical characteristics according to clinical outcome.

Variable	Categories	Recovered (*n* = 64)	LT (*n* = 107)	Death (*n* = 4)	*p*
Sex	Male	27 (42.2)	44 (41.1)	2 (50.0)	0.935
	Female	37 (57.8)	63 (58.9)	2 (50.0)	
LT Type	LDLT	0 (0.0)	77 (72.0)	0 (0.0)	—
	DDLT	0 (0.0)	30 (28.0)	0 (0.0)	
Pattern of liver injury *n* (%)	Hepatocellular	60 (93.8)	90 (84.1)	3 (75.0)	0.067
	Cholestatic	4 (6.3)	10 (9.3)	0 (0.0)	
	Mixed	0 (0.0)	7 (6.5)	1 (25.0)	
Hy’s law *n* (%)	Negative	22 (34.4)	9 (17.8)	1 (25.0)	0.048
	Positive	42 (65.6)	88 (82.2)	3 (75.0)	
Classical Hy’s law *n* (%)	Negative	30 (46.9)	47 (43.9)	2 (50.0)	0.914
	Positive	34 (53.1)	60 (56.1)	2 (50.0)	
DILIN-derived liver injury severity grade, *n* (%)	Mild	12 (18.8)	0 (0.0)	0 (0.0)	<0.001
	Moderate	34 (53.1)	11 (10.3)	1 (25.0)	
	Severe	18 (28.1)	96 (89.7)	3 (75.0)	
Groups, *n* (%)	HILI	16 (25.0	1 (0.9)	0 (0)	<0.001
	DILI	38 (59.4)	55 (51.4)	4 (100)	
	Mushroom poisoning	6 (9.4)	18 (16.8)	0 (0)	
	Suspected causative agent	4 (6.3)	11 (10.3)	0 (0)	
	NA	0 (0)	22 (20.6)	0 (0)	

**Table 7:** Comparison of continuous clinical and laboratory variables between recovery and liver transplantation- death.

Variables	Recovered (*n* = 64)	LT and Death (*n* = 111)	*p*
Age	40 (33–55)	25 (10–44)	<0.001
WBC	7 (6–9)	9 (7–13)	<0.001
Lymphocyte	1.8 (1.4–2.3)	1.3 (0.8–2.1)	0.002
Neutrophil	4.6 (3.5–5.4)	6.9 (3.7–9.6)	<0.001
Hemoglobin	13.6 (12.3–15.0)	12.0 (10.3–13.3)	<0.001
Platelet	221 (170–270)	202 (126–277)	0.378
AST	878 (519–1651)	1658 (699–3394)	<0.001
ALT	1057 (649–2090)	1385 (606–3942)	0.120
ALP	163 (102–218)	202 (134–282)	0.004
GGT	129 (56–225)	64 (46–122)	0.002
INR	1.4 (1.2–1.8)	3.4 (2.3–4.8)	<0.001
Total bilirubin	6.3 (1.9–16.5)	13.6 (5.8–22.0)	<0.001
Direct bilirubin	4.7 (1.0–11.5)	8.0 (3.7–13.0)	0.002
Albumin	3.5 (2.9–3.8)	3.1 (2.5–3.5)	<0.001
Ammonia	106 (88–186)	201 (138–431)	<0.001
Creatinine	0.7 (0.6–0.8)	0.6 (0.5–0.8)	0.010
Sodium	138 (136–139)	136 (133–140)	0.687
MELD Na	19 (14–25)	32 (27–36)	<0.001
R ratio	20.9 (11.7–40.3)	22.9 (9.1–47.4)	0.873

**Table 8:** Comparison of categorical clinical characteristics between recovery and liver transplantation- death.

Variable	Categories	Recovered (*n* = 64)	LT and Death (*n* = 111)	*p*
Sex	Male	27 (42.2)	46 (41.4)	0.923
	Female	37 (57.8)	65 (58.6)	
LT Type	LDLT	0 (0.0)	77 (72.0)	—
	DDLT	0 (0.0)	30 (28.0)	
Pattern of liver injury *n* (%)	Hepatocellular	60 (93.8)	93 (83.8)	0.065
	Cholestatic	4 (6.3)	10 (9.0)	
	Mixed	0 (0.0)	8 (7.2)	
Hy’s law *n* (%)	Negative	22 (34.4)	20 (18.0)	0.015
	Positive	42 (65.6)	91 (82.0)	
Classical Hy’s law *n* (%)	Negative	30 (46.9)	49 (44.1)	0.727
	Positive	34 (53.1)	62 (55.9)	
DILIN-derived liver injury severity grade, *n* (%)	Mild	12 (18.8)	0 (0.0)	<0.001
	Moderate	34 (53.1)	12 (10.8)	
	Severe	18 (28.1)	99 (89.2)	
Groups, *n* (%)	HILI	16 (25.0)	1 (0.9)	<0.001
	DILI	38 (59.4)	59 (53.2)	
	Mushroom poisoning	6 (9.4)	18 (16.2)	
	Suspected causative agent	4 (6.3)	11 (9.9)	
	NA	0 (0.0)	22 (19.8)	

**Table 9:** Multivariable logistic regression analysis of factors associated with recovery after toxic hepatitis.

Variables	B	SE	Wald	OR	95%CI	*p*
Age	0.064	0.019	11.332	1.066	1.03–1.11	0.001
AST	0.000	0.000	2.326	1.000	1.00–1.00	0.127
INR	−1.584	0.467	11.514	0.205	0.08–0.51	0.001
Tbil	−0.019	0.030	0.403	0.982	0.93–1.04	0.525
Albumin	1.450	0.515	7.929	4.255	1.55–11.63	0.005
Ammonia	−0.005	0.005	1.150	0.995	0.99–1.00	0.283
Creatinine	0.863	1.252	0.475	2.370	0.20–27.78	0.491

## Data Availability

The data presented in this study are not publicly available due to ethical and privacy restrictions. Anonymized data may be available from the corresponding author upon reasonable request and subject to institutional approval.

## Supplementary Materials

The following supporting information can be downloaded at: https://www.mdpi.com/article/10.3390/medicina62071290/s1, Table S1. Institutional reference ranges for laboratory parameters included in the study. Figure S1. Representative contrast-enhanced abdominal CT imaging example of acute toxic hepatitis.

## Abbreviations

The following abbreviations are used in this manuscript:

**A****bbreviation**	**Definition**
ACG	American College of Gastroenterology
ALF	Acute liver failure
ALP	Alkaline phosphatase
ALT	Alanine aminotransferase
AST	Aspartate aminotransferase
AUC	Area under the receiver operating characteristic curve
CI	Confidence interval
DDLT	Deceased donor liver transplantation
DILI	Drug-induced liver injury
DILIN	Drug-Induced Liver Injury Network
EASL	European Association for the Study of the Liver
GGT	Gamma-glutamyl transferase
HDS	Herbal and dietary supplements
HILI	Herbal product-related liver injury
INR	International normalized ratio
IQR	Interquartile range
KCC	King’s College Criteria
LDLT	Living donor liver transplantation
LT	Liver transplantation
MELD-Na	Model for End-Stage Liver Disease incorporating serum sodium
NA	Not available
OR	Odds ratio
PELD	Pediatric End-Stage Liver Disease
ROC	Receiver operating characteristic
RUCAM	Roussel Uclaf Causality Assessment Method
ULN	Upper limit of normal
WBC	White blood cell count
