# Supplementary material for: Etiology-Based Comparative Evaluation of Toxic Hepatitis in a Tertiary Referral Cohort: Drug-Induced, Herb-Induced, Mushroom Poisoning, and Other Toxic Exposures
Source: Medicina (Kaunas). 2026 Jul 3;62(7):1290. doi: 10.3390/medicina62071290 (PMC13413763; doi:10.3390/medicina62071290)
Supplement: Supplementary file 1 [file medicina-62-01290-s001.zip › medicina-4376225-supplementary.pdf]

Etiology-Based Comparative Evaluation of Toxic Hepatitis in a Tertiary Referral Cohort: Drug-Induced, Herb-Induced, Mushroom Poisoning, and Other Toxic Exposures

Suheda Rumeysa Osmanlioglu-Dag[1]*, Nurullah Dag[2]*, Sami Akbulut[3,4], Muhsin Murat Muhip Harputluoglu[5], Sezai Yılmaz[3]

[1] Department of Pharmaceutical Botany, Faculty of Pharmacy, Inonu University, 44280, Malatya, Türkiye
[2] Department of Radiology, Faculty of Medicine, Inonu University, 44280, Malatya, Türkiye
[3] Department of Surgery and Liver Transplantation, Faculty of Medicine, Inonu University, 44280, Malatya, Türkiye
[4] Department of Biostatistics and Medical Informatics, Faculty of Medicine, Inonu University, 44280, Malatya, Türkiye
[5] Department of Gastroenterology, Faculty of Medicine, Inonu University, 44280, Malatya, Türkiye

1* Correspondence: rumeysa.osmanlioglu@inonu.edu.tr
2* Correspondence: nurullah.dag@inonu.edu.tr

**Contents**	**Page**
**Table S1.** Institutional reference ranges for laboratory parameters included in the study	**2**
**Figure S1.** Representative contrast-enhanced abdominal CT imaging example of acute toxic hepatitis.	**3**

Table S1. Institutional reference ranges for laboratory parameters included in the study

Variable	Unit	Reference range
**WBC**	$\times 10^3/\mu L$	4.3–10.3
**Lymphocyte**	$\times 10^3/\mu L$	1.3–3.5
**Neutrophil**	$\times 10^3/\mu L$	2.1–6.1
**Hemoglobin**	g/dL	13.6–17.2
**Platelet**	$\times 10^3/\mu L$	156–373
**AST**	U/L	8–48
**ALT**	U/L	7–55
**ALP**	U/L	30–120
**GGT**	U/L	12–64
**INR**	ratio	0.9–1.2
**Total bilirubin**	mg/dL	0.2–1.2
**Direct bilirubin**	mg/dL	≤0.5
**Albumin**	g/dL	3.5–5.0
**Ammonia**	µg/dL	≤60
**Creatinine**	mg/dL	0.57–1.25
**Sodium**	mmol/L	136–145

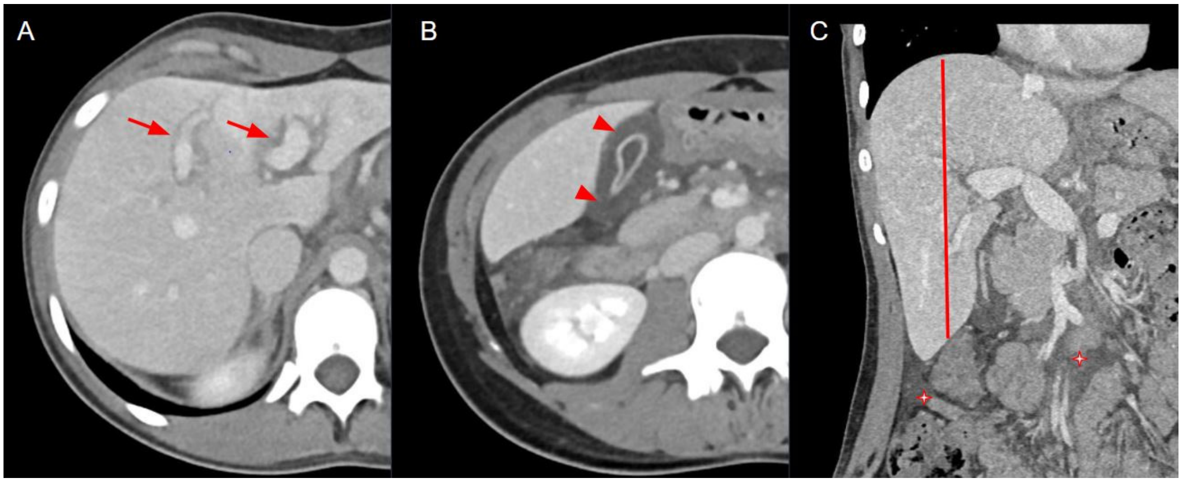

Figure S1. Representative contrast-enhanced abdominal CT imaging example of acute toxic hepatitis. Axial contrast-enhanced CT image (A) shows periportal edema, visible as low-attenuation periportal tracking (arrows), together with mild heterogeneous attenuation of the hepatic parenchyma. Axial contrast-enhanced CT image (B) demonstrates gallbladder wall thickening and edema (arrowheads). Coronal contrast-enhanced CT image (C) shows hepatomegaly and minimal intra-abdominal free fluid (stars). This figure is provided for illustrative purposes only; radiologic findings were not systematically evaluated or included in the statistical analyses.
